# A Case of Rapidly Progressing Hepatocellular Carcinoma after Administration of JAK Inhibitors to Treat Rheumatoid Arthritis

**DOI:** 10.1155/2022/6852189

**Published:** 2022-03-29

**Authors:** Rioko Migita, Yasutaka Kimoto, Junki Hiura, Yuta Okumura, Takahiko Horiuchi

**Affiliations:** Kyushu University Beppu Hospital, Oita, Japan

## Abstract

We report a case of rapidly progressing hepatocellular carcinoma after administration of Janus kinase (JAK) inhibitors to treat rheumatoid arthritis. A 76-year-old man was referred to our Department for pain in multiple joints and was diagnosed with rheumatoid arthritis. Blood tests revealed elevated hepatobiliary enzymes, but various tests revealed no signs suggestive of malignancy. He took baricitinib for 2 months followed by tofacitinib for 4 months. After that, he was diagnosed with hepatocellular carcinoma based on imaging findings and elevated tumor markers. This case showed the possibility of a causal relationship between JAK inhibitors and malignancy.

## 1. Introduction

The JAK-STAT pathway is associated with cytokine signaling, and JAK inhibitors suppress inflammation in rheumatoid arthritis [1]. In patients with rheumatoid arthritis that is poorly controlled by initial treatment with methotrexate (MTX) or other conventional synthetic disease-modifying antirheumatic drugs (csDMARDs), the European League against Rheumatic Diseases (EULAR) recommends considering the use of JAK inhibitors [2]. Five JAK inhibitors—tofacitinib, baricitinib, peficitinib, upadacitinib, and filgotinib—are currently approved in the area of rheumatoid arthritis.

At the current point in time, no studies have reported that use of a JAK inhibitor increases the risk of malignancy, but additional cases need to be assembled. We encountered a case where the JAK inhibitors baricitinib and tofacitinib were administered to treat rheumatoid arthritis and a large hepatocellular carcinoma developed. A potential relationship between JAK inhibitors and malignancy warrants discussion and has been reported here.

## 2. Case Presentation

**Figure 1:**
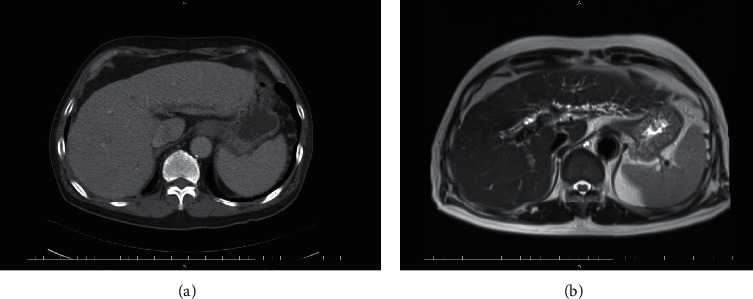
Contrast-enhanced CT (a) and contrast-enhanced MRI (b) did not show any sign of hepatic tumor.

**Figure 2:**
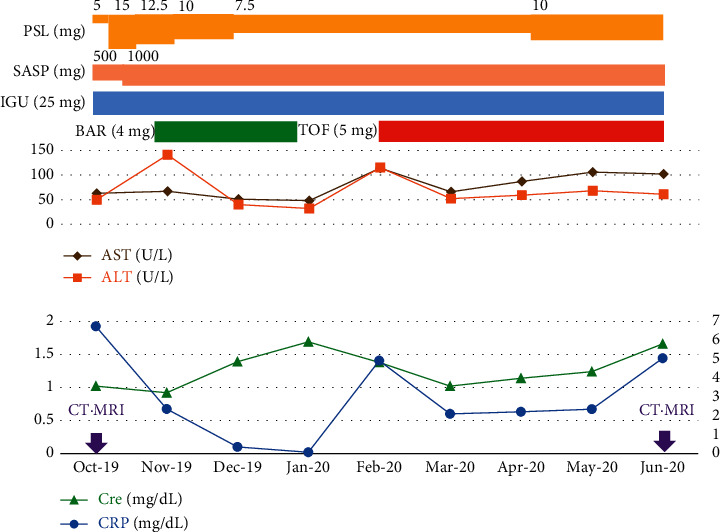
Clinical course of the patient. ALT: alanine aminotransferase; AST: aspartate aminotransferase; BAR: baricitinib; cre: creatinine; CRP: C-reactive protein; CT: computed tomography; IGU: iguratimod; MRI: magnetic resonance imaging; PSL: prednisolone; SASP: salazosulfapyridine; TOF: tofacitinib.

**Figure 3:**
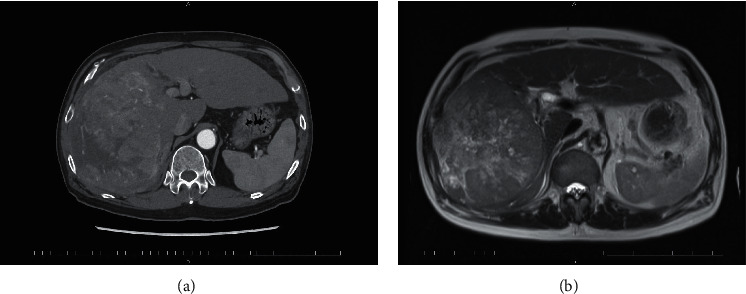
Most of the right lobe of the liver was occupied by a huge tumor (13 cm × 10 cm) on contrast-enhanced CT (a) and contrast-enhanced MRI (b).

The patient was a 76-year-old male. He had a history of hypertension and had a history of smoking and drinking alcohol. He was referred to our Department in October 2019 for pain in multiple joints and stiffness of the fingers in the morning. Swelling of multiple joints was noted upon initial examination, and both rheumatoid factor (RF) and anti-cyclic citrullinated peptides antibody (anti-CCP antibody) were positive (RF was 118.1 IU/mL and anti-CCP antibody titer was 938 U/mL). Inflammatory indices were also elevated, e.g., a sedimentation rate (in 1 hour) of 90 mm and a C-reactive protein (CRP) level of 6.74 mg/dL, so the patient was diagnosed with rheumatoid arthritis. Blood tests revealed elevated hepatobiliary enzymes, e.g., aspartate aminotransferase (AST) of 63 U/L, alanine aminotransferase (ALT) of 50 U/L, alkaline phosphatase (ALP) of 1,385 U/L, and *γ*-glutamyl transpeptidase (*γ*-GTP) of 368 U/L, but hepatitis B antigen and antibody tests and a hepatitis C antibody test were all negative. *α*-Fetoprotein (AFP) and protein induced by vitamin K absence or antagonist II (PIVKAII) were within the normal range. Total bilirubin was 0.30 mg/dL, and direct bilirubin was 0.08 mg/dL. Contrast-enhanced computed tomography (CT) and contrast-enhanced magnetic resonance imaging (MRI) revealed findings suspected of cirrhosis. These also revealed intrahepatic bile duct dilation and stenosis but no signs suggestive of malignancy (Figure 1). Child-Pugh classification was A. IgG4-related disease and primary biliary cholangitis were suspected, but IgG4 levels were within the normal range and anti-mitochondrial antibody was negative. Treatment with salazosulfapyridine (SASP) and prednisolone (PSL) was initiated, and iguratimod (IGU) was added. One month after the patient was diagnosed with rheumatoid arthritis, the patient began taking baricitinib 4 mg/day and continued to take that drug for 2 months. Renal dysfunction developed, so baricitinib was discontinued and the patient did not receive a JAK inhibitor for 1 month. The patient subsequently started taking tofacitinib 5 mg/day and continued to take that drug for 4 months (Figure 2). After JAK inhibitors were administered for 6 months (June 2020), blood test results were an AST of 102 U/L, an ALT of 61 U/L, an ALP of 594 U/L, and a *γ*-GTP of 427 U/L. Total bilirubin was 1.30 mg/dL, and direct bilirubin was 0.56 mg/dL. The patient underwent contrast-enhanced CT and contrast-enhanced MRI for follow-up, and those examinations revealed a large mass (10 cm × 13 cm) occupying most of the right lobe of the liver (Figure 3). In both examinations, enhancement was noted in the early phase of contrast enhancement and washout of the contrast agent was noted in the late phase. These are classic findings for hepatocellular carcinoma. Tumor markers were markedly elevated (AFP was 111,167 ng/mL and PIVKA-II was 971,000 mAU/mL). Based on these findings and imaging findings, the patient was diagnosed with hepatocellular carcinoma.

## 3. Discussion

JAK is a family of intracellular tyrosine kinases including JAK1, JAK2, JAK3, and TYK2, and the JAK family is closely associated with cytokine receptor signalling [3]. When cytokines bind to receptors, JAK is activated; as a result, STAT is phosphorylated and genetic programming specific to cytokines is activated [4]. Type I and type II cytokines, which include IL-6, GM-CSF, and IFN-*γ*, use the JAK-STAT pathway for signaling, so JAK inhibitors suppress inflammation in rheumatoid arthritis [1].

Tofacitinib is a JAK inhibitor that was initially approved for treatment of rheumatoid arthritis, and it primarily inhibits JAK1 and JAK3 [3]. Tofacitinib is mainly metabolized in the liver via cytochrome P450 (CYP) 3A4 [5]. Numerous phase III trials have indicated the efficacy of tofacitinib [6–9]. In contrast, baricitinib is a JAK1/JAK2 inhibitor that inhibits intracellular signaling by numerous inflammatory cytokines such as IL-6, IL-12, and IL-23 [10]. Unlike tofacitinib, baricitinib is primarily metabolized by the kidneys [11]. Numerous phase III trials have indicated the efficacy of baricitinib [12–14].

An open-label long-term study examined the safety of tofacitinib in 4,481 patients with rheumatoid arthritis who received either 5 mg or 10 mg twice a day for up to 9.5 years, but it found no increased risk of malignancy (which included hepatocellular carcinoma) [15]. Moreover, an analysis of data from clinical trials involving 6,194 patients receiving tofacitinib [16] and a 3-year postmarketing surveillance study of tofacitinib [17] revealed no increased risk of malignancy (which included hepatocellular carcinoma). An analysis of data from clinical trials involving 3,492 patients receiving baricitinib (the duration of administration was up to 5.5 years) revealed no increased risk of malignancy (which included hepatocellular carcinoma) [18]. No studies have reported that tofacitinib and baricitinib increase the risk of malignancy until recently, but it was recently reported that tofacitinib may increase the risk of malignancy. Pfizer Inc. (NYSE: PFE) announced co-primary endpoint results from a recently completed postmarketing-required safety study, ORAL Surveillance (A3921133; NCT02092467), on January 27, 2021. [19]. The co-primary endpoints of this study were noninferiority of tofacitinib compared to TNF inhibitors in regard to major adverse cardiovascular events (MACEs) and malignancies (excluding nonmelanoma skin cancer (NMSC)). Results showed that for these co-primary endpoints, the prespecified noninferiority criteria were not met for the primary comparison of the combined tofacitinib doses to TNFi.

A study reported that STAT3 is routinely activated in up to 60% of cases of hepatocellular carcinoma (HCC) [20]. STAT3 is associated with the proliferation of HCC cells; e.g., it promotes progression of the cell cycle [21] and it inhibits the expression of an apoptosis-promoting protein [22]. STAT3 is also associated with promotion of VEGF expression and promotion of angiogenesis [23]. STAT3 is also associated with promotion of stem cell-like properties in HCC cells [24] and promotion of the Warburg effect in cancer cells [25]. When JAK2 is inhibited, IL-6-mediated activation of STAT3 is inhibited. Although they were both in the preclinical stage, one study reported that the JAK2 inhibitor pacritinib reduced hepatic fibrosis in a mouse model [26] while another reported that the JAK1/JAK2 inhibitor ruxolitinib inhibited cell proliferation and colony formation by HCC cell lines [27].

Activated STAT1 inhibits angiogenesis by suppressing tumor formation [28], and STAT2 represses the transcription of an oncogene [29], but the specific role of STATs in HCC has not yet been determined. A study reported that STAT4 is expressed at a significantly lower level in HCC than in normal tissue [30], but the specific role of STAT4 and STAT5 in HCC is also unclear. Tofacitinib, a JAK1/JAK3 inhibitor, is associated with inhibition of STAT5 activation, but the relationship between STAT5 and HCC needs to be clarified further.

When hepatocytes become cancerous, membrane-bound MHC class I polypeptide-related sequence A (MICA) is expressed on the cell surface, inducing an attack by natural killer (NK) cells [31]. This is how the innate immune system eliminates cancerous cells. However, membrane-bound MICA is cleaved from tumor cells, resulting in a soluble form that binds to NK cells and that can evade immune surveillance by NK cells [32]. Tofacitinib is associated with inhibition of IL-15, and IL-15 is associated with the differentiation and maintenance of NK cells, so tofacitinib may be associated with risk of malignancy because it reduces the number of NK cells.

In this case, elevated hepatobiliary enzymes were noted when the patient was diagnosed with rheumatoid arthritis, but blood tests and imaging studies revealed no signs suggestive of malignancy. Contrast-enhanced CT revealed findings suspected of cirrhosis and he had a history of drinking alcohol, so it is considered that there was a risk of developing hepatocellular carcinoma. The patient was subsequently administered baricitinib for 2 months and tofacitinib for 4 months, whereupon contrast-enhanced CT revealed a large hepatic tumor. Given the classic imaging findings for hepatocellular carcinoma and high levels of tumor markers, the patient was diagnosed with hepatocellular carcinoma.

There are many aspects of the relationship between tofacitinib and baricitinib and malignancy that have yet to be clarified. Tofacitinib was recently reported to have the potential to increase the risk of malignancy, but its causal relationship with hepatocellular carcinoma is unclear. In this case, the causal relationship between JAK inhibitors and hepatocellular carcinoma cannot be determined because the patient originally had risk factors for hepatocellular carcinoma. However, it is possible that JAK inhibitors contributed to the development of hepatocellular carcinoma in the present case. The relationship between JAK inhibitors and malignancy needs to be elucidated further. We suggest that careful follow-up is required when administering JAK inhibitors.
